# Molecular and Replication Dynamic Profiling of Regionally Important *Pestivirus bovis* Subgenotypes from Hungary

**DOI:** 10.3390/ani16071106

**Published:** 2026-04-03

**Authors:** István Kiss, Eszter Kaszab, Krisztina Bali, Renáta Varga-Kugler, Scott Callison, Derek E. Moormeier, Liliana Cubas-Gaona, Zalán Homonnay, Krisztián Bányai

**Affiliations:** 1 Ceva-Phylaxia Ltd., 1107 Budapest, Hungary; renata.varga-kugler@ceva.com (R.V.-K.); lcubas.gaona@gmail.com (L.C.-G.); zalan.homonnay@ceva.com (Z.H.); 2 Department of Microbiology and Infectious Diseases, University of Veterinary Medicine, 1078 Budapest, Hungarybali.krisztina@univet.hu (K.B.); 3 HUN-REN Veterinary Medical Research Institute, 1143 Budapest, Hungary; bkrota@hotmail.com; 4 Department of Bioinformatics, One Health Institute, University of Debrecen, 4032 Debrecen, Hungary; 5 National Laboratory for Infectious Animal Diseases, Antimicrobial Resistance, Veterinary Public Health and Food Chain Safety, 1078 Budapest, Hungary; 6 NGS & Bioinformatics Platform, Ceva Animal Health, Shawnee, KS 66215, USA; scott.callison@ceva.com (S.C.); derek.moormeier@ceva.com (D.E.M.); 7 Innovation R&D, BIOGENOVAC-CEVA Beaucouzé Campus, 49070 Beaucouzé, France; 8 Department of Pharmacology and Toxicology, University of Veterinary Medicine, 1078 Budapest, Hungary; 9 Department of Medical Biology, Medical School, University of Pécs, 7624 Pécs, Hungary

**Keywords:** biotype, BVDV, kinetic, subgenotype, substitutions

## Abstract

This study investigated the *in vitro* replication kinetics and molecular characteristics of five field isolates of bovine viral diarrhea virus (BVDV) representing subgenotypes 1b, 1d, and 1f, currently circulating in Hungary. We compared cytopathogenic (cp) and non-cytopathogenic (ncp) biotype pairs using digital PCR (dPCR) and virus titration. While dPCR showed higher genome copy numbers for cp isolates, virus titration revealed comparable or lower infectious titers, suggesting the accumulation of replication-incompetent viral particles during the infection cycle. Molecular analysis identified (novel) amino acid substitutions in Npro, capsid, and NS4B regions, although typical large-scale genome rearrangements were absent. These findings demonstrate that biotype differences are molecularly complex and subgenotype-dependent. Our results emphasize that relying on a few genetic markers is insufficient for biotype categorization, necessitating comprehensive characterization in BVDV surveillance programs. This complexity must be considered when designing vaccines or control programs, especially in regions with diverse circulating strains.

## Simple Summary

Bovine viral diarrhea is an economically significant cattle disease. A common, non-cytopathogenic biotype of the virus can spontaneously change into a form that kills cells and causes severe disease. We showed here subtle differences in viral replication kinetics in laboratory settings. Mutational changes in the genome previously associated with the virulent biotype were not identified, but novel, small-scale genetic substitutions were recorded. These findings show that the switch between biotypes is a complex process and provide new potential markers for diagnostics, valuable information to develop improved disease control and prevention measures.

## 1. Introduction

Bovine viral diarrhea (BVD), caused by pestiviruses, is a globally significant cattle disease characterized by high genetic diversity [1]. The strains within a highly prevalent pestivirus species, *Pestivirus bovis*, are classified into subgenotypes, mainly based on partial 5′-UTR, Npro and E2 coding sequences [2]. The most prevalent subgenotypes are represented in commercial vaccines, either as mono- or bivalent products [3]. Importantly, vaccines of broad cross-immunization characteristics provide a highly efficient and versatile asset to the control of BVD caused by viruses with diverse genetic backgrounds [4,5].

Bovine viral diarrhea virus (BVDV) strains are categorized into cytopathogenic (cp) and non-cytopathogenic (ncp) biotypes based on cell culture propagation. The cp biotype evolves from ncp strains through genomic rearrangements—such as duplications, cellular sequence insertions, deletions, and point mutations—and induces more extensive host gene expression changes in cell culture, particularly within innate immune and metabolic regulatory pathways [6,7,8].

Due to the direct and indirect impact of BVD, a number of countries introduced eradication programs to control the disease and the circulation of the virus [9]. Previously, we reported on a recent investigation on the circulating BVDV strains and the eradication campaign conducted successfully in Hungary [10,11]. Our findings demonstrated the presence of subgenotype 1b, 1d, and 1f *Pestivirus bovis* strains in the country, representing the most widespread subgenotypes over Europe [2].

Stronger replication capacities are expected to provide transmission advantages to the viruses, potentially by reaching higher viral load, increased shedding rate, and shorter incubation period. Furthermore, rapid replication can lead to faster evolution and adaptation, consequently, selecting for these variants to overcome host immune responses more efficiently. Therefore, obtaining this information on the currently circulating strains is of particular importance.

Functional comparisons between different BVDV subgenotypes and biotypes are currently limited. Therefore, the aim of this study was to perform a comparative analysis of representative viral isolates. This analysis focused on two key aspects: their replication kinetics *in vitro* and their molecular composition. The goal was to identify differences among biotypes and locally prevalent subgenotypes, thereby contributing to a better understanding of their disease-causing potential.

## 2. Materials and Methods

### 2.1. Virus Isolation and Titration

The viruses used in the study (a cp/ncp pair of subgenotype 1f and 1b, and a ncp isolate of subgenotype 1d) originated mainly from serum samples collected from clinically healthy calves throughout the voluntary BVD eradication program pursued in Hungary since 2020 [10,11] and are listed in Table 1. The 1b cp strain was collected from a calf showing severe diarrhea, depression, and erosions in the palate, before it perished due to its deteriorating condition.

The MDBK cells used in the study originated from the cell-line inventory of Ceva-Phylaxia, Budapest, Hungary, and were used for primary virus isolation from 5× diluted serum samples, and from 1:10 tissue homogenate in PBS, filtered through 0.22 µm sterile filter (Merck Millipore Ltd., Tullagreen, Carrigtwohill, Co., Cork, Ireland), in 1:1 mixture of MEM-H and MEM-E medium (Gibco, Thermo Fisher Scientific, Waltham, MA, USA), on Falcon T25 cell culture flasks (Corning Inc., Corning, NY, USA), at 37 °C in a 5% CO_2_ atmosphere. The cultures were checked daily for CP effect, and four–five days post-inoculation were harvested.

For the kinetic investigations, MDBK cells were infected at a multiplicity of infection (MOI) of 1.0 in 2 × 7 (parallel testing) 25 mL flasks per strain. MOI calculation was based on the titers determined by the Reed and Muench method [12]. The inocula were back-titrated. The cells were frozen at −20 °C at 0 h, 8 h, 24 h, 48 h, 72 h, 96 h, and 120 h post-infection. Virus titers in cell lysate (after ultrasound homogenization) and supernatant at each timepoint were determined as described by Reed and Muench [12]. The ncp strains were visualized by immunofluorescent staining. The titrations were visualized by immunofluorescent staining using a pestivirus-specific monoclonal antibody mix of murine origin as the primary antibodies, followed by fluorescein isothiocyanate (FITC)-conjugated anti-mouse IgG. Fluorescence was detected at an emission wavelength of approximately 520 nm.

### 2.2. Digital PCR (dPCR)

Viral RNA was extracted with MagMax™ Express 96 instrument (Thermo Fisher Scientific, Waltham, MA, USA) using the IndiMag Pathogen kit (Indical Bioscience, Leipzig, Germany) according to the manufacturer’s protocol. Viral load was quantified using the QIAcuity Digital PCR System (Qiagen, Hilden, Germany) with pan-pestivirus RT-PCR primers and probe published by Hoffmann and co-workers [13]. The reaction was performed in a 12 µL reaction volume (4 µL of extracted RNA) in triplicate on a Nanoplate 8.5k with QIAcuity OneStep Advanced Probe RT-dPCR reaction mix (Qiagen) according to the manufacturer’s instructions and using the forward primer:BVD190-F GRAGTCGTCARTGGTTCGAC; reverse primer: V326 TCAACTCCATGTGCCATGTAC; and probe: TQ-Pesti TGCYAYGTGGACGAGGGCATGC (all presented 5′ → 3′). The reverse transcription was carried out at 50 °C for 40 min, followed by an inactivation step at 95 °C for 2 min. The amplification had 40 cycles of 95 °C for 5 s and 60 °C for 30 s.

### 2.3. Whole Genome Sequencing and Analysis

Isolates from 2021 were whole-genome sequenced on an Illumina platform as described previously [14]. Isolates from 2023 were sequenced by Oxford Nanopore sequencing as follows: RNA was extracted using ZymoBIOMICS DNA/RNA Miniprep kit (Zymo Research, Irvine, CA, USA) with DNase treatment, included in the kit. The host rRNA was depleted with Invitrogen RiboMinus Eukaryote System v2 (Thermo Fisher Scientific, Waltham, MA, USA). REPLI-g WTA Single Cell Kit was used for reverse transcription and cDNA amplification. The library was prepared with a Rapid Barcoding Kit 24 V14 (Oxford Nanopore Technologies, Oxford, UK) and was sequenced on R10.4.1 flowcell in a MinION Mk1B sequencing device.

For taxonomic classification, the raw sequence files were analyzed via the Kaiju webserver v1.10.1 (database NCBI nr + euk) [15] or with an in-house proprietary data analysis pipeline. Complete genome sequences from short and long reads were assembled via mapping to the reference AF091605 GenBank sequence using Geneious Prime^®^ v.2022.2.2 (Biomatters, Auckland, New Zealand, https://www.geneious.com).

The polyprotein CDS and individual coding gene sequences were predicted with the ORF finder tool (https://www.ncbi.nlm.nih.gov/orffinder/ accessed on 1 September 2025) and with Geneious Prime^®^ v.2022.2.2 software. Nucleotide (nt) and amino acid (aa) sequences of polyprotein and individual coding genes were aligned by the MAFFT plugin of Geneious Prime^®^ v.2022.2.2. The obtained sequences were deposited in GenBank; accession numbers are as follows: PV554201-05.

### 2.4. Area Under the Curve (AUC) Calculation

Area under the curve (AUC) calculations were performed using GraphPad Prism version 10.3, applying the trapezoidal rule to compare viral yields between biotype counterparts. For each sample, the mean of log_10_-transformed titers obtained from parallel titrations was used in the analysis. The variability among replicates was utilized to estimate the standard error (SE) of the AUC and its corresponding 95% confidence interval (CI).

To assess statistical uncertainty in AUC differences between biotypes, a nonparametric bootstrap with 10,000 iterations was implemented by resampling timepoints with replacement and recalculating AUCs for each resample. Bootstrap 95% confidence intervals (CI) were derived from the percentile method (e.g., ΔAUC_1_ CI = 8.4–47.6; ΔAUC_1β_ CI = −81.6 to −18.8).

To test whether cp and ncp curves were exchangeable under the null hypothesis, a two-sided permutation test was conducted by randomly swapping cp/ncp labels within each timepoint across 10,000 iterations. *p*-values were calculated as the proportion of permuted AUC differences exceeding the observed effect in absolute magnitude (e.g., *p* = 0.093 for dataset 1f; *p* = 0.0491 for dataset 1b).

These nonparametric approaches were selected because they do not assume normality and are well-suited for growth-curve comparisons derived from limited biological sample sizes.

## 3. Results

### 3.1. Analysis of Viral Kinetics in Cell Culture

**Figure 1:**
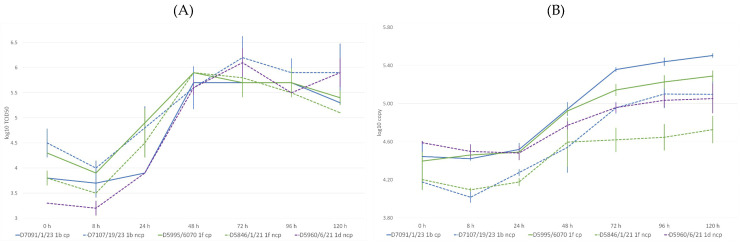
Panel (**A**): Measured virus titers over time in hours (h). Data refers to the average of two replicates. Panel (**B**): Measured virus copy numbers over time in hours (h). Data refers to the average of two test points.

The initially determined titers and copy numbers showed some degree of diversity at MOI 1.0 (Figure 1A,B). Specifically, higher initial values were observed for the 1f cp strain than its ncp counterpart (log 4.4 and 3.8 TCID_50_/0.1 mL, respectively), and, on the contrary, higher for the 1b ncp strain than for its cp counterpart (log 4.5 and 3.8 TCID_50_/0.1 mL).

Virus titration revealed a drop in viral titer at 8 h post-infection. By 24 h, titers began to rise, reaching a peak at 48 h for the cytopathogenic (cp) viruses. This was followed by a plateau lasting until 96 h, and a decline by 120 h (Figure 1). Among the non-cytopathogenic (ncp) strains, 1b and 1d peaked at 72 h and showed a slight decrease by 120 h. Interestingly, the 1f ncp strain displayed replication kinetics more similar to the cp viruses. Notably, the 1d ncp strain replicated at a slower rate than the other viruses during the first 24 h.

The dPCR indicated a difference between the kinetics of the ncp and cp viruses, the latter reaching higher copy numbers over 120 h. The initial copy number was higher for the 1d ncp virus than for the other two of the same biotype. The 1f ncp strain consistently demonstrated the lowest number of copies.

Comparison of growth kinetics between cp and ncp biotype counterparts revealed isolate-specific differences in cumulative viral output. For isolate pair 1f, the cp variant displayed a slightly higher overall replication, with AUC values of 642.0 (cp) and 621.2 (ncp), yielding an observed ΔAUC of 20.8 log_10_·h. Bootstrap resampling produced a 95% CI of 8.4–47.6 log_10_·h, while the permutation test indicated a statistical trend (*p* = 0.093), suggesting modest but consistent cp-associated elevation in viral yield. In contrast, isolate pair 1b showed the opposite pattern; AUC values were 611.6 (cp) and 655.2 (ncp), corresponding to a ΔAUC of −43.6 log_10_·h, with a bootstrap 95% CI of −81.6 to −18.8 and a significant permutation *p*-value of 0.0491. Together, these analyses demonstrate that while AUC-based differences were detectable in both datasets, the direction and magnitude of biotype effects varied by isolate, with pair 1b showing a statistically robust disadvantage for the cp variant.

### 3.2. Molecular Characterization of Viral Strains

All three strains of 2021 origin were sequenced by the Illumina-based methodology. Strain D5995/6070/21/1f/cp/4 was sequenced by both Illumina and ONT platforms, and since there was practically no difference between the outcome of the analyses, yielding 8000–9000X coverage, the 2023 isolates were sequenced by the ONT platform only. The obtained sequences were of the following nucleotide lengths: D5846/2/21/1f/ncp/3—12,214 nt; D5995/6070/21/1f/cp/4—12,255 nt; D5960/6/21/1d/ncp/3—12,130 nt; D7107/19/23/1b/ncp/4—12,096 nt; and D7091/1/23/1b/cp/4—12,179 nt.

**Figure 2:**
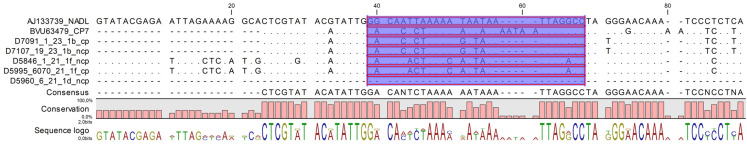
Partial 5′-UTR sequences of the analyzed viruses. The sequence fragment encompassing the Ib hairpin loop is highlighted. The 1a subgenotype NADL and CP7 strains are used as references. Dots indicate identical nucleotides.

**Figure 3:**
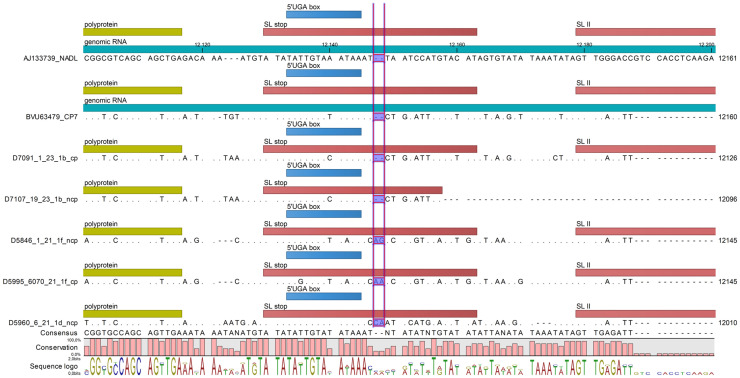
Partial 3′-UTR sequences of the analyzed viruses. The very end of the polyprotein coding region (green), as well as the SL stop (dark red), the UGA box (blue), and the beginning of the SLII (coral) regions, are indicated. The differing 2 nucleotides, or their lack of difference, are highlighted.

Concerning the untranslated regions, only the 1f pair yielded the near-complete sequences of both the 5′ and 3′ UTRs. The obtained sequences for the hairpin Ib of the 5′-UTR [16] indicated differences between the 1b and 1f viruses, but not between the related biotypes (Figure 2). The presence of the highly conserved AGCACUUUA nt stretch was detected from the 3′-UTR, i.e., the two 1f and the 1d ncp strains. The SL stop region of the 1b pair contained a 2 nt deletion compared to the 1f and 1d viruses (Figure 3).

The coding regions of all five strains were determined and analyzed. The observed amino acid differences in the coding regions are summarized in Table 2. The 1d ncp strain was used as a basis for the molecular analysis, as it does not have a cp counterpair. The largest amino acid difference between the polyproteins was 13% and observed between the 1f ncp and 1d ncp strains, affecting 510 amino acids. The ncp/cp counterparts demonstrated a 9 (1b) and 29 (1f) amino acid differences on the whole coding region. The nucleocapsid coding sequence of the 1d subgenotype virus had its 10th amino acid deleted compared to the rest of the strains analyzed.

Further analysis of the biotype-specific differences revealed no insertions or partial genome duplications in the two cp isolates between the NS2-3 coding region. Mutations affecting the highly conserved NS4B 2441 (residue 15 of NS4B) amino acid position play a crucial role in BVDV cytopathogenicity, with Y2441 representing the “reference” variant [17]. All strains included in our study shared the Y2441 amino acid. However, the NS4B K28E and S89T differentiated the 1b and 1f ncp/cp counterparts, respectively.

## 4. Discussion

Investigating *in vitro* replication kinetics provides essential data regarding viral transmission potential and is crucial for the design and optimization of vaccine production. While *in vitro* rates are not fully predictive of *in vivo* outcomes, a fast replication rate and high virion output may indicate increased transmission risk. While ncp BVDV strains represent the predominant form of the virus in field conditions, cp variants are considered evolutionary ‘dead-ends’ for both the virus and the host [18]. Historically, cp biotype viruses have been observed to exhibit faster replication rates than ncp variants [19,20]. Our findings demonstrate that the kinetic profiles of these Hungarian field isolates are influenced not only by their biotype but also by their specific subgenotype.

The diversity observed in initial titers and copy numbers at the same MOI reflects infectivity differences between isolates, where higher values indicate more efficient replication capabilities [21]. Statistical validation using AUC analysis indicated that the kinetic differences can be influenced by the biotype, the subgenotype and/or the initial infectious titers of individual strains. In fact, the biotype-associated replication advantages were not uniform across subgenotypes in our small dataset, and the observed divergence suggests that viral ‘fitness’ is highly isolate-specific, which may be constrained by the genetic background. This preliminary insight into replication kinetics of local BVDV strains could affect the design and optimization of vaccine production, although a better understanding and resolution of the whole issue make large-scale studies involving additional strains necessary. Nonetheless, the findings presented here offer valuable insights that could help guide molecular analyses, particularly when comparing replication kinetics.

A notable finding was the discrepancy between dPCR and virus titration. While dPCR indicated higher genome copy numbers for cp viruses at later stages, titration revealed rather uniform infective quantities for both biotypes. This suggests the presence of more ‘non-viable’ viral genomes during these periods. BVDV exists as a viral quasispecies, a phenomenon demonstrated both *in vivo* and *in vitro* [22,23]. While this aspect fell beyond the scope of this study, our observations are suggestive of a thriving viral quasispecies within the cp biotype, comprising an increasing volume of replication-incompetent variants [24]. The specific expression patterns of these variants may contribute to the cytopathic effect [8]. Potential mechanisms include a higher rate of replication accompanied by deleterious mutations or interference with cellular signaling pathways [25]. Discriminating between viable and non-viable viral genomic material is essential when evaluating vaccine efficacy, as vaccine-induced immune responses must effectively reduce virus replication and transmission [26,27,28,29].

When comparing BVDV genomes and deduced protein sequences, we arbitrarily chose the subgenotype 1d strain as a baseline, and greater amino acid diversity was observed among the 1f subgenotype strains than among their 1b counterparts. Furthermore, compared to other subgenotypes, a single amino acid deletion was observed at position 10 in the nucleocapsid coding region of the 1d strain. A BLAST search of 1d subgenotype sequences from China, Brazil, and Japan confirmed this finding (GenBank accession numbers: KT951840, KT951841, MG923683, AB359927, and AB359928). Determining if this deletion in various 1d strains plays a role in the observed replication kinetics of the relevant isolate would be an intriguing investigation.

Molecular characterization revealed that the investigated cp isolates lacked typical large-scale genome rearrangements, such as insertions of cellular sequences or duplications in the NS2-3 region [6,7]. Instead, accumulated point mutations likely drive cytopathogenicity in these strains [30]. For instance, while the NS4B Y2441C substitution is known to reconstitute the ncp biotype in the NADL strain [17], all our isolates shared the “reference” Y2441 amino acid. Similarly, at position 1555—a key site for cytopathogenicity in the Oregon C24V strain [31]—all investigated strains harbored phenylalanine (F) instead of serine (S). Given that the S → F substitution reduced NS2-3 cleavage efficiency by 50% in the Oregon strain, the biotype of these Hungarian viruses must be determined by alternative factors. Of interest, in some cp viruses, NS2-3 is not processed but translated from defective interfering genomes [31]. Furthermore, instead of large genome rearrangements such as insertion of viral or cellular sequences or genome duplications, accumulated point mutations can also be responsible for cytopathogenicity [30].

Structural proteins, particularly E2, showed the greatest amino acid diversity, confirming they are most prone to variability due to host immune pressure. We analyzed Npro and capsid coding sequences as they are associated with biotype-determining functions [32]. While the type 1b pair showed no amino acid differences in these regions, a single substitution (A18V) was noted in the type 1f pair. Npro, along with Erns, plays an important role in evading host immune responses and establishing persistent infection [33]. Furthermore, a unique amino acid deletion in the nucleocapsid was identified in the 1d subgenotype isolate. While this molecular signature has been documented in 1d strains globally, a better assessment of its functional role in replication kinetics warrants further targeted study and cannot be definitively linked to regional adaptation.

The untranslated regions (UTRs) of the BVDV genome play a vital role in replication kinetics [16,34,35,36,37] and serve as a basis for pestivirus classification [38]. In the 5′-UTR, the distribution of A residues within the Ib loop, as well as the 5′-GUAU motif, may impact replication capabilities [16]. In the 3′-UTR, the conserved AGCACUUUA motif within the SS region was detected in all strains where sequencing was successful [36]. The SL stop region confirmed high variability, consistent with its characterization as an unstable, A-U-rich segment [39].

## 5. Conclusions

While this study provides data to link replication dynamics, biotypes, and genomic data, essential for understanding the pathobiology and evolution of regionally important *Pestivirus bovis* subgenotypes, it is constrained by a small sample size (n = 5). Nonetheless, this research establishes a genomic and phenotypic baseline for regional isolates. Broader studies may reveal whether the same or similar patterns occur elsewhere. Overall, the findings reinforce that BVDV biotypes exhibit substantial molecular complexity and cannot be fully characterized using traditional genetic markers alone.

## Figures and Tables

**Table 1:** Virus isolates used in the study. Primary subgenotype determination was based on Npro coding sequences.

Sample ID/Subgenotype/Biotype/Passage #	Farm ID	Sample	Collection date	Sequencing Platform	Obtained nt Length
D5846/2/21/1f/ncp/3	Y	serum	06/2021	Illumina	12,214
D5995/6070/21/1f/cp/4	Y	serum	08/2021	Illumina, ONT	12,255
D5960/6/21/1d/ncp/3	D	serum	07/2021	Illumina	12,130
D7107/19/23/1b/ncp/4	N	serum	03/2023	ONT	12,096
D7091/1/23/1b/cp/4	N	ovarium	03/2023	ONT	12,179

**Table 2:** Amino acid differences related to D5960/6/21 1d ncp strain and between the respective biotype viruses.

Number of aa Differences to D5960/6/21/HU 1d/ncp	D7107/19/23 1b ncp	D7091/1/23 1b cp	1b ncp/cp Substitutions	D5846/1/21 1f ncp	D5995/6070 1f cp	1f ncp/cp Substitutions
Polyprotein	430 (88.97) †	435 (88.84)	-	501 (87.15)	510 (86.92)	-
N^pro^	23 (86.31)	23 (86.31)	-	28 (83.33)	29 (82.47)	A18V
E^rns^	19 (91.67)	19 (91.67)	-	22 (90.35)	24 (89.47)	M51T, E86G, A172V, V208K, K211R
Core	14 (86.54)	15 (85.58)	K55R	18 (82.69)	20 (80.77)	D47E, K50R
E1	29 (84.90)	29 (84.90)	M185I	28 (85.42)	28 (85.42)	-
E2	88 (76.47)	88 (76.47)	S144N, P191L, Y339F	89 (76.20)	92 (75.40)	H1Y, G50E, A159T, V190I, G239D, K272R
p7	14 (80.00)	14 (80.00)	-	12 (82.86)	12 (82.86)	-
NS2	70 (84.55)	73 (83.89)	T335A, K358E, H387Y	79 (82.56)	78 (82.78)	T110A, N363S
NS3	12 (98.24)	12 (98.24)	-	21 (96.93)	21 (96.93)	-
NS4A	2 (96.88)	2 (96.88)	-	3 (95.31)	3 (95.31)	-
NS4B	21 (93.95)	20 (94.24)	E28K	22 (93.66)	23 (93.37)	S89T
NS5A	80 (83.87)	80 (83.87)	-	90 (81.85)	93 (81.25)	T23A, M24V, L51S, S70N, D180N, I260M, N366D
NS5B	59 (91.79)	59 (91.79)	-	88 (87.76)	88 (87.76)	N88S, K105R, S164N, E664G, R672K

† Percentile sequence similarity values are shown in parentheses.

## Data Availability

Datasets generated during the study can be found at https://doi.org/10.6084/m9.figshare.27725436.v1. The GenBank accession numbers of the analyzed sequences are as follows: BankIt2951046 D5846/2/21/1f/ncp PV554201 (https://www.ncbi.nlm.nih.gov/nuccore/PV554201 accessed on 1 September 2025); BankIt2951046 D5960/6/21/1d/ncp PV554202 (https://www.ncbi.nlm.nih.gov/nuccore/PV554202 accessed on 1 September 2025); BankIt2951046 D5995/6070/21/1f/c PV554203 (https://www.ncbi.nlm.nih.gov/nuccore/PV554203 accessed on 1 September 2025); BankIt2951046 D7091/1/23/1b/cp PV554204 (https://www.ncbi.nlm.nih.gov/nuccore/PV554204 accessed on 1 September 2025); BankIt2951046 D7107/19/23/1b/ncp PV554205 (https://www.ncbi.nlm.nih.gov/nuccore/PV554205 accessed on 1 September 2025).
